# Case Report: Disseminated actinomycosis-induced splenic rupture with spleen and liver abscesses

**DOI:** 10.3389/fmed.2025.1654793

**Published:** 2025-09-05

**Authors:** Huaijuan Li, Zhaoping Cheng, Kui Li

**Affiliations:** ^1^Department of Infectious Diseases, Ankang Central Hospital, Ankang, China; ^2^Department of Pathology, Ankang Central Hospital, Ankang, China

**Keywords:** actinomycosis, complications, splenic rupture, abscess, case report

## Abstract

Disseminated actinomycosis is a rare, slowly progressing infection caused by Actinomyces species and can manifest as the formation of multiple abscesses and sulfur granules in infected tissues. In this report of this rare disease, the pathogens were not traced at the initial stage, and extremely rare but dangerous complications occurred, resulting in a new research perspective for the early identification of this rare disease. This study reports a case of disseminated actinomycosis in a 59-year-old Asian female with a history of lung infection. She experienced splenic rupture after mild percussion performed due to abdominal pain, accompanied by fever and sepsis; the rupture was effectively managed with conservative treatment; however, she experienced delayed splenic rupture later. Pathological examination of spleen and liver biopsy samples revealed chronic suppurative inflammation with abscesses, and the surrounding hyphae were arranged radially, the metagenome next-generation sequencing of pus shows that it belongs to *Actinomyces israelii*. The patient was cured with splenectomy and comprehensive treatment, including anti-infective therapy combined with imipenem/cilastatin, penicillin and antishock therapy. The results of this study emphasize the importance of etiology tracing and accurate, early treatment for the disease. Moreover, if minor trauma leads to unexpected damage to internal organs, the possibility of the coexistence of other etiologies should be considered.

## Introduction

Human infection with Actinomyces species was first reported in the 1880s. Actinomyces are gram-positive, filamentous, and facultatively anaerobic bacilli that are opportunistic pathogenic zoonotic bacteria. The characteristics of tissues infected with Actinomyces include purulent and granulomatous inflammation accompanied by sinus formation and sulfur granule discharge (1, 2). Actinomyces infections have an annual incidence of 1/300000 (3) and are well known for their complications. Because the imaging manifestations are not sufficiently specific to differentiate infection with Actinomyces from malignant tumors and other types of granulomatous inflammation, it is also known as an “outstanding impostor” and is associated with a preoperative diagnostic rate of less than 10% (4); thus, it is prone to misdiagnosis and can lead to adverse outcomes. Disseminated actinomycosis is an even rarer form of infection by Actinomyces, involving multiple organs and potentially manifesting as the formation of multiple abscesses in multiple locations and local symptoms. Although multiple clinical manifestations of disseminated actinomycosis have been reported, this disease remains poorly understood.

This study reports a case of disseminated actinomycosis complicated with splenic and liver abscesses. In the early stage, splenic rupture occurred after mild percussion, and the situation improved after antibiotics and conservative treatment. However, due to an inadequate course of anti-infective therapy, another spontaneous splenic rupture occurred, accompanied by sepsis, shock and impaired consciousness. Pathological examination of spleen and liver biopsy samples revealed chronic suppurative inflammation with abscesses, and the surrounding hyphae were arranged radially. The patient was diagnosed with disseminated actinomycosis and was cured after splenectomy, intensive care, and penicillin treatment. Splenic abscesses with rupture caused by actinomycosis is an extremely rare condition (5, 6), but recurrent rupture has not been reported. Anti-infective treatment is initially effective, but the patient subsequently experiences recurrence; thus, the possibility of actinomycosis should be considered. The key to avoiding poor patient outcomes is to focus on early pathogen tracing and to provide a full course of treatment with sensitive antibiotics.

## Case description

**FIGURE 1:**
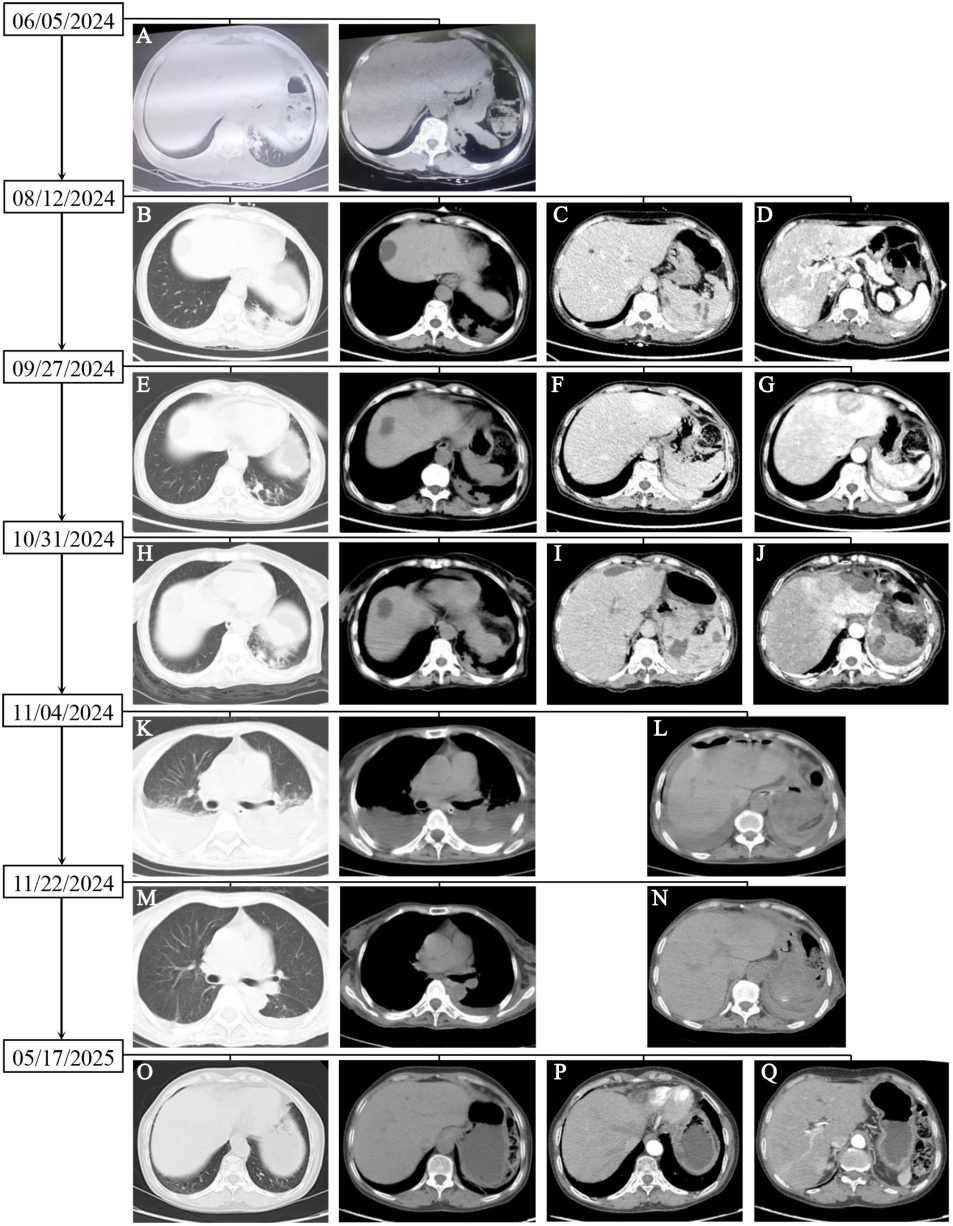
Dynamic changes in imaging examination results. **(A)** Inflammation of the left lower lung; **(B)** enlargement of the pneumonic lesion in the lower left lung; **(C)** splenic rupture on delayed-phase contrast-enhanced CT imaging; **(D)** abnormal perfusion of the S6 segment of the liver on arterial-phase contrast-enhanced CT imaging; **(E)** reduction in the lesion in the left lower lung; **(F)** the lesions in the spleen appear to have been essentially absorbed on delayed-phase contrast-enhanced CT imaging; **(G)** patchy low-density shadow in the left lobe of the liver; **(H)** unobvious changes in the lesions in the left lower lung; **(I)** enlarged lesions in the spleen on delayed-phase contrast-enhanced CT imaging; **(J)** slight enlargement of the lesions in the left outer lobe of the liver; **(K)** new inflammatory lesions in the lower lung and enlargement of the left pleural effusion; **(L)** slight reduction of the lesions at the junction of liver segments S2 and S4a; **(M)** significant absorption and reduction of pneumonic lesions in both lungs and pleural effusion: **(N)** slight reduction of the lesion at the junction of the S2 and S4a segments; **(O)** a few cord shadows in the left lower lung; **(P)** reduction of the lesions at the junction of hepatic segments S2 and S4a; **(Q)** poor visualization of lesions in hepatic segment S6.

The patient was a 59-year-old Asian female with intermittent cough, abdominal pain, and a maximum temperature of 39.2 °C. She reported a slight cough approximately 2 months prior. Chest computed tomography (CT) revealed pneumonia and atelectasis in the left lower lobe (Figure 1A). The patient had been prescribed oral medication for several days at a local hospital, the specifics of which were unknown, but the intermittent cough persisted. The abdominal pain appeared without obvious symptoms 10 days prior, mainly in the upper abdomen, but was tolerable. Gastroenteroscopy did not reveal obvious abnormalities. Later, she tapped herself with a hard object due to the abdominal pain, which then worsened with abdominal distension. Because color ultrasound examination at a local hospital revealed splenic trauma and a small amount of fluid around the spleen, the patient was transferred to our hospital for further diagnosis and treatment. At the time of admission, her vital signs were stable, and her body mass index (BMI) was 17.22 kg/m^2^. She had tenderness in the abdomen but no rebound tenderness. Other examinations did not reveal significant abnormalities. The routine blood examination revealed that a white blood cell (WBC) count of 8.97 × 10^9^/L, a hemoglobin (Hb) level of 96 g/L, and a procalcitonin level of 1.564 ng/ml. Blood cultures were normal. Non-contrast-enhanced CT scans of the head and pelvis revealed no abnormalities. Non-contrast-enhanced CT scans of the chest revealed inflammation in the lower lobes of both lungs and a small amount of left pleural effusion (Figure 1B). Non-contrast-enhanced and contrast-enhanced CT scans of the abdomen suggested splenic rupture (Figure 1C) and abnormal perfusion of the S6 segment of the liver on arterial-phase imaging (Figure 1D). On the basis of these findings, the patient was diagnosed with splenic rupture and bilateral lung infections. Fever developed the next day, and the patient received an intravenous injection of cefuroxime sodium (1.5 g/time every 8 h) for 1 week as anti-infective treatment. Reexamination showed an Hb level of 103 g/L. CT scans revealed that the splenic lesion had grown and that a small amount of perisplenic fluid had been absorbed. The symptoms were relieved after 10 days of conservative treatment, and the patient was discharged from the hospital. After discharge, the patient continued to receive cefuroxime sodium for 10 days, after which the treatment was discontinued; however, the patient still experienced mild abdominal pain. Six weeks after discharge, a follow-up chest CT scan revealed that the lesions in the left lower lung had shrunk (Figure 1E). Abdominal CT revealed that the lesions in the spleen were essentially absorbed (Figure 1F), and a patchy low-density shadow was observed in the left lobe of the liver. Contrast-enhanced CT scans revealed significant enhancement of the center of the lesion, with delayed enhancement on the edges (Figure 1G).

Sixty-nine days after discharge, the patient was hospitalized again due to abdominal pain for 5 h, with persistent colic. The physical examination results were as follows: temperature, 39.3 °C; blood pressure, 82/46 mm Hg; digital pulse oxygen saturation, 98%; random blood glucose, 6.2 mmol/L; acute facial pain; and obvious tenderness in the upper abdomen. The change in the left lower lung lesion on the chest CT scan was not obvious (Figure 1H). Non-contrast-enhanced and contrast-enhanced CT scans of the abdomen revealed that, compared with their prior appearances, the spleen was enlarged and presented with more lesions (Figure 1I), and the lesions at the junction of liver segments S2 and S4a and liver segment S3 were slightly enlarged (Figure 1J). After emergency treatment in the emergency department of internal medicine, the patient was transferred to general surgery the next day for splenectomy, space-occupying resection of the left lateral lobe of the liver, and biopsy. Intraoperatively, the greater omentum was seen to have gathered toward the left upper quadrant (LUQ), blood had accumulated in the LUQ, pus and moss had adhered to the greater omentum and spleen, a 3-cm irregular fissure with active hemorrhage was seen at the lower pole of the spleen, and two nodules were seen in the left outer lobe of the liver, the larger of which was approximately 4 cm in diameter.

**FIGURE 2:**
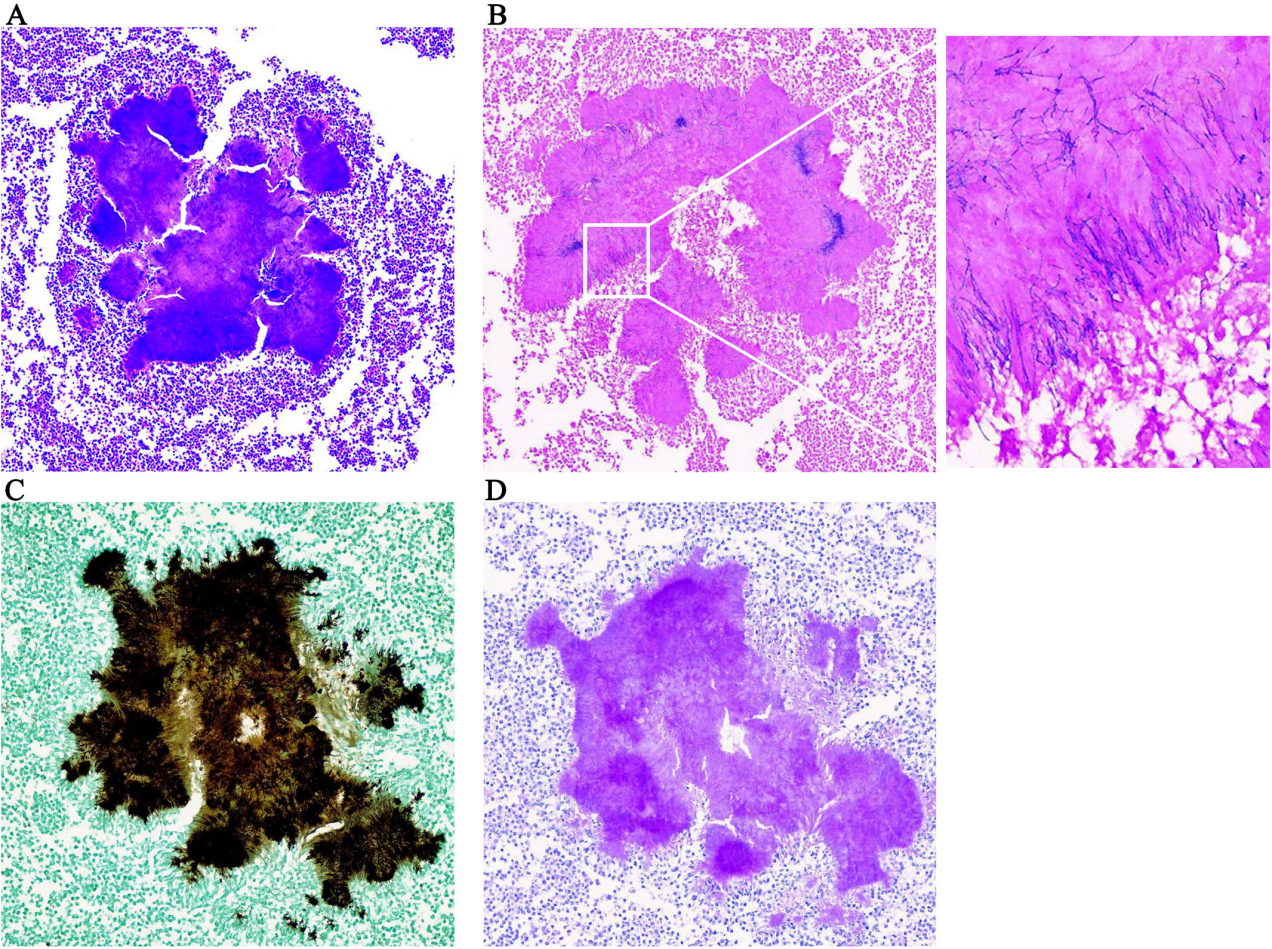
Pathological examination results. **(A)** HE × 200; **(B)** positive gram staining × 200; the enlarged image shows radial arrangement of blue filamentous bacterial colonies; **(C)** positive hexamine silver staining × 200; **(D)** positive PAS-D staining × 200.

The clinical and imaging manifestations of actinomycosis are relatively non-specific, and the positive culture rate is low. Gram staining of pus and pathological examination of infected tissues are highly important for the diagnosis of actinomycosis and are usually more sensitive than culture (4). Pathological examination of the two resected tissues of this patient (spleen and liver) suggested chronic suppurative inflammation; cell clusters could be observed in the abscesses, and the surrounding hyphae were arranged in a radial pattern. Following amylase digestion, Gram, silver hexamine and periodic acid-Schiff-diastase (PAS-D) staining were positive and acid-fast staining was negative (Figure 2), the metagenome next-generation sequencing (mNGS) of pus shows that it belongs to *Actinomyces israelii* (High signal strength, sequence number 675), confirming the diagnosis of disseminated actinomycosis. Actinomycosis needs to be differentiated from a malignant tumor. Although imaging examinations are relatively convenient, actinomycosis and malignant tumors both demonstrate enhancement, so an imaging diagnosis is challenging. Conformation of the diagnosis ultimately depends on the valuable information provided by culture, histopathological staining and molecular biology.

**FIGURE 3:**
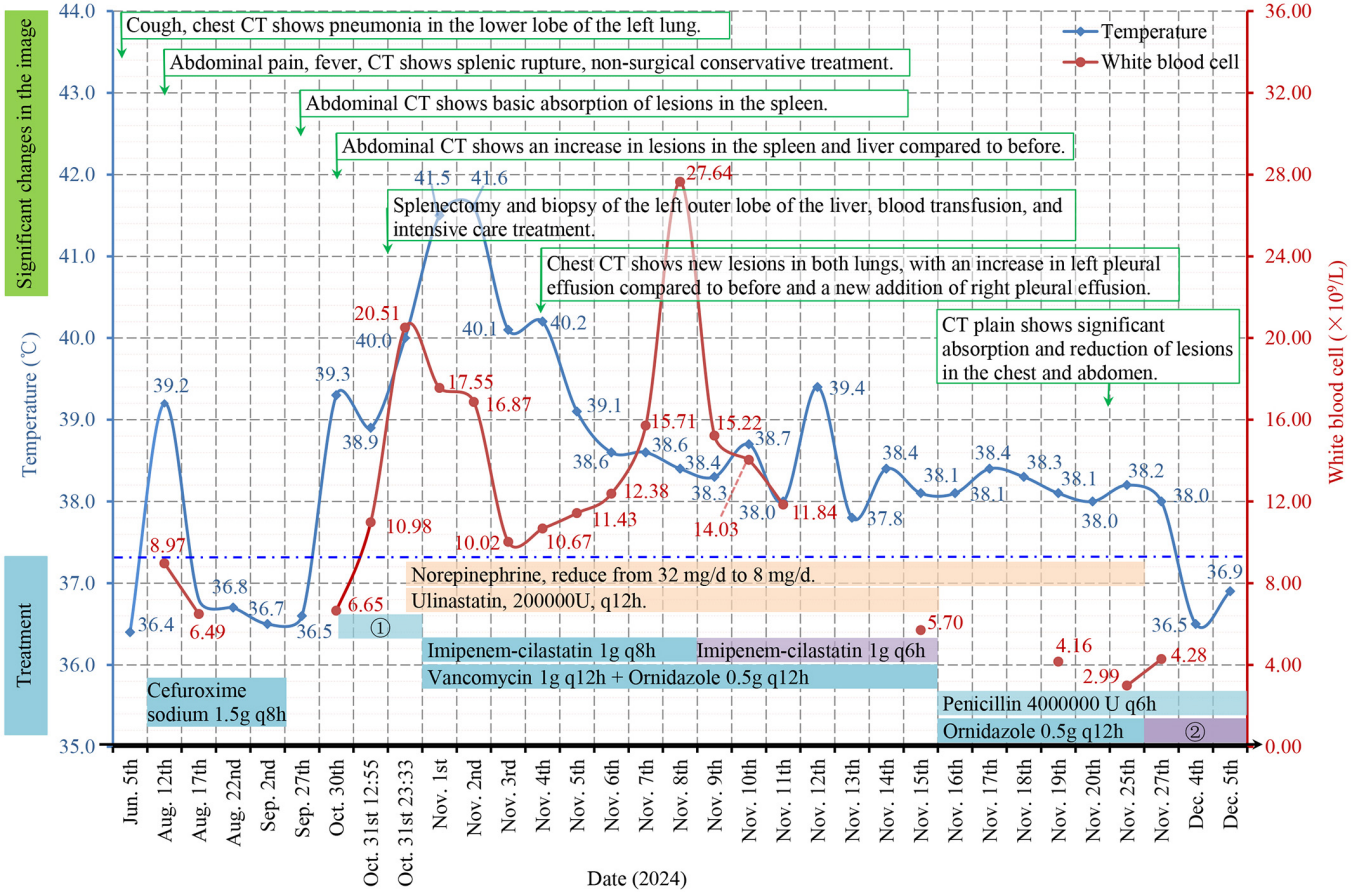
Timeline of the clinical course. The clinical findings were associated with significant changes in the imaging results and treatment of the patient. ? Cefuroxime sodium was used 2 times, and cefoperazone sodium sulbactam sodium (2.0 g) combined with ornidazole (0.5 g) was used once preoperatively; ? Cefoperazone sodium/sulbactam sodium 1.5 g q12h; q(x)h: the dose is depicted as once every × hours; the temperature in the figure is the highest measured value among several temperature measurements on the same day; the dotted blue line represents the normal upper limit of human axillary temperature.

The patient had persistent hypotension (75/45 mmHg) in the middle and late parts of the operation. After surgery, the patient was lethargic, with clammy limbs and profuse sweating. The patient was transferred to the intensive care unit (ICU) with a very high fever (41.5 °C) and given the comprehensive treatment, including imipenem-cilastatin sodium, vancomycin, and ornidazole for combined anti-infective treatment, vasoactive drugs (norepinephrine, dobutamine) to maintain her blood pressure, ulinastatin for anti-inflammatory treatment, plasma and serum albumin transfusion for hepatoprotection and anticoagulation (starting on the 5th day after surgery), cardiotonics, and diuretics. On the 4th day after surgery, non-contrast-enhanced CT scans of the chest revealed new lesions in both lungs, increased left pleural effusion, and a new right pleural effusion (Figure 1K). CT scans of the upper abdomen revealed that the lesion at the junction of the S2 and S4a segments was slightly smaller than before, the amount of subcapsular fluid in the left lobe of the liver had decreased, and a small amount of blood (fluid) had accumulated in the surgical area of the spleen (Figure 1L). Drainage of the pleural effusion fluid was performed, revealing exudate changes, and cultures of the effusion fluid showed no bacterial growth. A second blood culture was negative. The patient’s consciousness improved on the 6th day after surgery. On the 8th day after surgery, the WBC count significantly increased, and the intensity of antibiotic use increased. However, the patient still had a moderate fever daily. The patient was transferred from the ICU to the Department of Infectious Diseases 15 days after surgery, where she was given penicillin combined with ornidazole. The third blood culture was negative. Non-contrast-enhanced CT scans of the chest obtained 22 days after surgery revealed that the pneumonic lesion and pleural effusion were significantly absorbed and reduced in size (Figure 1M). Abdominal CT revealed that the lesions at the junction of liver segments S2 and S4a were slightly smaller than before, the lesion in liver segment S6 was basically unchanged, the subcapsular fluid of the left liver lobe and the amount of fluid ascites in the spleen area were reduced, and the pneumoperitoneum was basically absorbed (Figure 1N). After norepinephrine was discontinued 25 days after surgery, her blood pressure fluctuated around 100/65 mmHg, and she did not have a fever again. During the disease course, there was no vegetation formation observed in three echocardiograms, platelet counts and alanine/aspartate aminotransferase, procalcitonin, and C-reactive protein levels significantly increased once, then gradually returned to normal after the patient received the corresponding treatments. The specific changes are shown in Supplementary Table 1. Figure 3 summarizes the changes in clinical and imaging characteristics over the disease course following implementation of important therapeutic measures.

After she was discharged from the hospital, the patient was followed up with telephone calls and home visits. The patient had no further fever or abdominal pain. The anti-infective treatments continued for 6 months, including oral amoxicillin-clavulanate potassium (0.375 g 3 times/d) and sulfamethoxazole (0.96 g 3 times/d). The results of routine blood tests and liver function tests were essentially normal. Non-contrast-enhanced CT scans of the chest revealed a few cord shadows in the left lower lung (Figure 1O), and contrast-enhanced CT scans of the upper abdomen revealed that the lesion at the junction of liver segments S2 and S4a was smaller than before (Figure 1P), while the lesion at the S6 segment of the liver could not be clearly displayed (Figure 1Q). As of the writing of this manuscript, the patient has gained 5 kg, can take care of herself completely, and can tolerate routine labor.

## Discussion

Actinomyces are non-spore-forming, capsular, flagellar, anaerobic-microaerophilic filamentous bacilli that stain negative for acid-fast stain and positive for Gram stain (7). These bacilli are opportunistic pathogens that form a part of the normal flora of the human oral cavity, gastrointestinal tract and female urogenital tract. Among them, six species are considered pathogenic for humans, including *Actinomyces israelii*, *Actinomyces naeslundii*, *Actinomyces odontolyticus, Actinomyces viscous*, *Actinomyces meyeri*, and *Actinomyces gerencseriae*. *A. israelii* is the most common pathogen of human diseases, and *A. meyeri* may be more likely to affect the lungs and blood transmission, but this transmission tendency is difficult to explain because the pathogen does not differ from other species in terms of microbial characteristics (2).

Actinomyces are not toxic bacilli. When breaking through the mucosal barrier, the pathogens can secrete a protective biofilm, sulfur granules can inhibit phagocytosis and evade attack by the host immune system (2), cell wall lipoproteins induce an excessive immune response through Toll-like receptors, causing pathogens to expand beyond the mucosa (8, 9), and cell wall peptidoglycan can induce bone resorption, osteoclastogenesis and the recruitment of inflammatory cytokines, thus causing purulent and granulomatous inflammation (10). Infection can lead to the formation of abscesses and sinus tracts and can continuously spread to any organ without the restriction of the tissue barrier. Moreover, the infection is multibacterial and may coexist with infection by Fusobacteria, Bacteroides, Enterobacteriaceae and other bacteria depending on the infection site; Actinomyces can be accompanied by 2–4 species on average, sometimes as many as 10 species (8, 11). Ayantunde et al. (12) reported that mixed microorganisms were isolated in 57.2% of the samples collected. These companion bacteria may play a role in the infection by creating an anaerobic environment in which Actinomyces thrives, reducing oxygen tension in the tissues to inhibit phagocyte function or producing toxins that promote the growth of Actinomyces (13–15). Some studies have suggested that this phenomenon might be caused by a mixed infection of a variety of bacteria dominated by Actinomyces (16, 17).

The most common site of infection is the face and neck, accounting for approximately 50%–60% of all infections. Abdominal actinomycosis is rare, accounting for only 20% of all Actinomyces infections, while infection in the lungs accounts for 15% (1). Two-thirds of cases of abdominal actinomycosis involve the appendix and cecum (18), and studies have shown that 3/4 of these cases are caused by perforation due to appendicitis (19). Abdominal surgery (20) and implantation of an intrauterine device (IUD) (21) are the most important risk factors for abdominal actinomycosis. The age of onset in this case is highly similar to the mean age (58 ± 12 years) reported by Zhang et al. (14), but none of the aforementioned risk factors were the cause; instead, the fact that she has never practiced any oral hygiene measures since childhood may be one of the causative factors of her infection. When the patient was first admitted to the hospital, a lesion was present in the lower left lung. We speculated that the actinomycosis might have originated in the lungs, spread locally to the spleen, and then to the liver along the portal vein. The presence of relatively mild lung lesions but a severe degree of disease spread may provide new clues for the diagnosis of actinomycosis.

Actinomyces are fastidious bacteria that are difficult to culture, which requires brain heart infusion (bhi) agar, microaerophilicity, 37 °C, and 6%–10% CO_2_ for optimal growth. After 3–7 days of culture, “molar,” “raspberry” or “breadcrumb” colonies can be observed, in which case the observation time should be extended to 21 days. Species classification is of little use in guiding clinical treatment. The use of antibiotics before culture, even a single dose, may inhibit growth (22). Even if actinomycosis is highly suspected clinically, bacteriologically confirmed diagnosis is usually obtained in <50% of pathologically diagnosed cases (23). For suspected actinomycosis, when collecting a lung sample, the sample may be exposed to prolonged periods of aerobic conditions (>20 min) via the normal bronchoalveolar lavage method, which may result in false-negative cultures (24, 25); therefore, a protective brush should be used for anaerobic collection and examination (26). When performing CT or color Doppler ultrasound-guided puncture and drainage of abdominal lesions, attention should also be given to anaerobic conditions when samples are to be subjected to culture. In the pathological examination of tissue samples, sulfur granules are usually a feature of actinomycosis, but this cannot completely confirm the diagnosis because sulfur granules are also rarely found in nocardiosis, chromomycosis, *Prototheca segbwema* infections, mycotic tumor-like conditions, and *Staphylococcus* infections (17, 27). Molecular biology detection is a new technology used to diagnose actinomycosis. mNGS is highly sensitive, does not depend on anaerobic requirements of culture, and useful for the diagnosis of actinomycosis (28). Additionally, it does not depend on bacterial culture, and is highly cost- and time-effective. The mNGS can isolate and identify the ribonucleic acid or DNA of microorganisms and is a safe method for assessing microorganisms in samples (29).

The main principle of treatment is the long-term use of large doses of intravenous penicillin. Penicillin (18 million–24 million units per day) is typically used for 2–6 weeks, after which it (or amoxicillin) is administered orally for 6–12 months. Lung actinomycosis may require longer treatment times (20), while timely surgical removal of the lesion may be beneficial for shortening the course of antibiotics; however, clinical data supporting this approach are lacking. Alternative treatments for patients with penicillin allergies include ceftriaxone, doxycycline, macrolides and carbapenems (30). Ineffective drugs include aminoglycosides, metronidazole, aztreonam, compound sulfamethoxazole tablets, cephalexin, ceftazidime and antifungal drugs (31, 32). The combined use of metronidazole and β-lactamase inhibitors can also treat various microbial infections (8). There is still disagreement on whether concomitant mixed microorganisms should be considered when designing the initial anti-infective regimen. Cefuroxime sodium was used when the patient was first hospitalized, and the splenic abscesses improved over time but then ruptured again after worsening, suggesting the importance of long-term treatment. During the second hospitalization, after the use of carbapenems, the WBC count significantly increased, then gradually recovered after the intensity of the antibiotics increased. However, the clinical response was delayed, and the temperature returned to normal 10 days after the use of penicillin, suggesting that carbapenems are not superior to penicillin in the treatment of actinomycosis. Moreover, nitroimidazole antibiotics were used throughout the entire process on the basis of empirical medication for the treatment of abdominal infections caused by pathogens.

The complications of actinomycosis include osteomyelitis, nervous system involvement, and endocarditis. This patient developed septic shock during surgery and required intensive care, which suggests the importance of management by a multidisciplinary team that includes infectious disease, intensive care, and general surgery personnel.

Most patients have a favorable outcome, but treatment may lead to extensive scar formation at the site of the lesion. Good oral hygiene, limited alcohol consumption, and replacement of any IUDs every 5 years may be beneficial for preventing Actinomyces infections.

The diagnosis and treatment process of this case also had certain limitations. For example, (1) the empirical anti-infective treatment for early pulmonary lesions was effective, but overlooking the etiological examination may have led to a missed diagnosis of the primary disease at the initial visit; (2) the occurrence of disseminated infection caused relevant checks of the patient’s immune status to be neglected. Although Actinomyces can cause abscesses in the liver and spleen, no current literature reports recurrent spleen rupture it induces. This presents a new research perspective for the early identification of this rare disease.

## Conclusion

Actinomyces infections demonstrate diverse clinical manifestations and atypical imaging features and are a rare cause of splenic abscess rupture. Gram staining of pus samples and pathological diagnoses of infected tissues are usually more sensitive than culture, while mNGS technology has greater diagnostic potential. Emphasis on early pathogen tracing and full-course treatment with sensitive antibiotics are key to avoiding poor patient outcomes.

## Data Availability

The original contributions presented in this study are included in this article/Supplementary material, further inquiries can be directed to the corresponding author.

## References

[1] Boot M Archer J Ali I. The diagnosis and management of pulmonary actinomycosis. *J Infect Public Health.* (2023) 16:490–500. 10.1016/j.jiph.2023.02.004 36801629

[2] Mabeza G Macfarlane J. Pulmonary actinomycosis. *Eur Respir J.* (2003) 21:545–51. 10.1183/09031936.03.00089103 12662015

[3] Boyanova L Kolarov R Mateva L Markovska R Mitov I. Actinomycosis: a frequently forgotten disease. *Future Microbiol.* (2015) 10:613–28. 10.2217/fmb.14.130 25865197

[4] Valour F Sénéchal A Dupieux C Karsenty J Lustig S Breton P Actinomycosis: etiology, clinical features, diagnosis, treatment, and management. *Infect Drug Resist.* (2014) 7:183–97. 10.2147/IDR.S39601 25045274 PMC4094581

[5] Sperling R Heredia R Gillesby W Chomet B. Rupture of the spleen secondary to actinomycosis. *Arch Surg.* (1967) 94:344–8. 10.1001/archsurg.1967.01330090038009 6018886

[6] Kakkasseril J Cabanas V Saba K. Ruptured actinomycotic aneurysm of the splenic artery: a case report of successful resection. *Surgery.* (1983) 93:595–7. 6687642

[7] Kim S Jung L Oh I Kim Y Shin K Lee M Pulmonary actinomycosis during the first decade of 21st century: cases of 94 patients. *BMC Infect Dis.* (2013) 13:216. 10.1186/1471-2334-13-216 23672372 PMC3658925

[8] Sharma S Hashmi M Valentino ID. *Actinomycosis.* Treasure Island, FL: StatPearls Publishing (2023).

[9] Endo S Murayama F Yamaguchi T Yamamoto S Otani S Saito N Surgical considerations for pulmonary actinomycosis. *Ann Thorac Surg.* (2002) 74:185–90. 10.1016/s0003-4975(02)03616-0 12118755

[10] Bates M Cruickshank G. Thoracic actinomycosis. *Thorax.* (1957) 12:99–124. 10.1136/thx.12.2.99 13442954 PMC1019236

[11] Holm P. Studies on the aetiology of human actinomycosis. II. Do the other microbes of actinomycosis possess virulence? *Acta Pathol Microbiol Scand.* (1951) 28:391–406. 10.1111/j.1699-0463.1951.tb03705.x 14856745

[12] Ayantunde A Kiang J Raja N Ahmed J Sanghera A Venkatesha S Actinomyces species as emerging pathogens: an observational study of clinical infections and microbiological implications. *Cureus.* (2025) 17:e77128. 10.7759/cureus.77128 39925515 PMC11805605

[13] Gomes-Silva W Pereira D Fregnani E Almeida O Armada L Pires F. Clinicopathological and ultrastructural characterization of periapical actinomycosis. *Med Oral Patol Oral Cir Bucal.* (2020) 25:e131–6. 10.4317/medoral.23247 31880281 PMC6982982

[14] Zhang Y Shao C Sun Y Xu K Li J Huang H The clinical features and prognosis of 32 cases of pulmonary actinomycosis. *Zhonghua Jie He He Hu Xi Za Zhi.* (2020) 43:665–9. 10.3760/cma.j.cn112147-20200523-00627 32727178

[15] Palm A Isaksson J Brandén E Hillerdal G. Thoracal actinomycosis - a diagnostic challenge. *Lakartidningen.* (2019) 116:FR6A. 31821519

[16] Schaal K Lee H. Actinomycete infections in humans–a review. *Gene.* (1992) 115:201–11. 10.1016/0378-1119(92)90560-c 1612438

[17] Brown J. Human actinomycosis. A study of 181 subjects. *Hum Pathol.* (1973) 4:319–30. 10.1016/s0046-8177(73)80097-8 4756858

[18] Deshmukh N Heaney S. Actinomycosis at multiple colonic sites. *Am J Gastroenterol.* (1986) 81:1212–4. 3788934

[19] Orr K. Actinomycosis. *Br Med J.* (1973) 2:664. 10.1136/bmj.2.5867.664 4714860 PMC1589638

[20] Garner J Macdonald M Kumar P. Abdominal actinomycosis. *Int J Surg.* (2007) 5:441–8. 10.1016/j.ijsu.2006.06.009 18078685

[21] Hernández-Alonso R Ferrer Vilela I Fernández Del Castillo Ascanio M Pérez Álvarez AD Jordán Balanzá JC. Pelvic actinomycosis secondary to an intrauterine device. *Rev Esp Enferm Dig.* (2024) 116:714–6. 10.17235/reed.2023.10077/2023 38031924

[22] Tanaka-Bandoh K Watanabe K Kato N Ueno K. Susceptibilities of Actinomyces species and Propionibacterium propionicus to antimicrobial agents. *Clin Infect Dis.* (1997) 25:S262–3. 10.1086/516187 9310699

[23] Bennhoff D. Actinomycosis: diagnostic and therapeutic considerations and a review of 32 cases. *Laryngoscope.* (1984) 94:1198–217. 10.1288/00005537-198409000-00013 6381942

[24] Kinnear W MacFarlane JT. A survey of thoracic actinomycosis. *Respir Med.* (1990) 84:57–9. 10.1016/s0954-6111(08)80095-9 2371423

[25] Lee C Lin M Tsai Y Tsao T Lan R Chiang Y. Thoracic actinomycosis–review of 9 cases. *Changgeng Yi Xue Za Zhi.* (1991) 14:246–52. 1797368

[26] Smego R Foglia G. Actinomycosis. *Clin Infect Dis.* (1998) 26:1255–61. 10.1086/516337 9636842

[27] Wong V Turmezei T Weston V. Actinomycosis. *BMJ.* (2011) 343:d6099. 10.1136/bmj.d6099 21990282

[28] Wang W Ren D Xu C Yuan Q Zhang Q Hu H Pulmonary actinomycosis diagnosed by radial endobronchial ultrasound coupled with metagenomic next-generation sequencing: a case report and brief literature review. *Int J Infect Dis.* (2020) 100:379–81. 10.1016/j.ijid.2020.09.1418 32979589

[29] Ai J Li Y Cheng Q Cui P Wu H Xu B Diagnosis of local hepatic tuberculosis through next-generation sequencing: smarter, faster and better. *Clin Res Hepatol Gastroenterol.* (2018) 42:178–81. 10.1016/j.clinre.2018.04.007 29759945

[30] Song J Park H Jeon K Um S Kwon O Koh W. Treatment of thoracic actinomycosis: a retrospective analysis of 40 patients. *Ann Thorac Med.* (2010) 5:80–5. 10.4103/1817-1737.62470 20582172 PMC2883202

[31] Lerner P. Susceptibility of pathogenic actinomycetes to antimicrobial compounds. *Antimicrob Agents Chemother.* (1974) 5:302–9. 10.1128/AAC.5.3.302 4840438 PMC428965

[32] Smith A Hall V Thakker B Gemmell C. Antimicrobial susceptibility testing of Actinomyces species with 12 antimicrobial agents. *J Antimicrob Chemother.* (2005) 56:407–9. 10.1093/jac/dki206 15972310

