# Supplementary material for: Case Report: Disseminated actinomycosis-induced splenic rupture with spleen and liver abscesses
Source: Front Med (Lausanne). 2025 Sep 5;12:1654793. doi: 10.3389/fmed.2025.1654793 (PMC12446004; doi:10.3389/fmed.2025.1654793)
Supplement: Supplementary file 1 [file Table_1.docx]

| **Table 1** Blood routine, liver function, and inflammatory index testing results during the course of the disease | | | | | | | | |
| --- | --- | --- | --- | --- | --- | --- | --- | --- |
| Date  (2024) | Red blood corpuscle  (×10^12^/L) | Hemo-  globin  (g/L) | Platelet  (×10^9^/L) | Albumin  (g/L) | Alanine trans-  aminase (U/L) | Aspartate trans-  aminase  (U/L) | Procalci-  tonin  (ng/ml) | C-reactive protein (mg/L) |
| Aug. 12th | 3.35 | 96 | 277 | 31.7 | 27.8 | 40.0 | 1.564 | 93.58 |
| Aug. 17th | 3.64 | 103 | 368 | 29.9 | 27.2 | 35.7 | - | 38.15 |
| Oct. 30th | 3.70 | 96 | 573 | 35.8 | 18.8 | 29.6 | 7.800 | 189.95 |
| Oct. 31st 12:55 | 2.94 | 77 | 390 | 27.0 | 13.9 | 23.9 | 47.980 | - |
| Oct. 31st 23:33 | 3.42 | 96 | 224 | 21.8 | 23.4 | 90.0 | 38.470 | 192.65 |
| Nov. 1st | 3.17 | 89 | 267 | 28.3 | 22.1 | 54.4 | 32.540 | 227.13 |
| Nov. 2nd | 2.96 | 84 | 207 | 34.8 | 578.6 | 2415.2 | 16.830 | 197.48 |
| Nov. 3rd | 3.06 | 84 | 225 | 32.1 | 677.2 | 1204.4 | 10.260 | 137.42 |
| Nov. 4th | 3.42 | 95 | 244 | 30.0 | 371.3 | 285.6 | 5.351 | 71.42 |
| Nov. 5th | 4.42 | 121 | 318 | 31.5 | 302.0 | 288.6 | 2.129 | 38.22 |
| Nov. 6th | 4.47 | 122 | 349 | 28.8 | 232.2 | 228.5 | 1.709 | 27.75 |
| Nov. 7th | 3.92 | 109 | 392 | 29.2 | 185.1 | 205.1 | 0.789 | 43.35 |
| Nov. 8th | 3.81 | 107 | 432 | - | - | - | - | 47.00 |
| Nov. 9th | 3.95 | 113 | 498 | 30.0 | 113.0 | 91.9 | 0.745 | 62.01 |
| Nov. 10th | 3.72 | 106 | 319 | 24.4 | 83.1 | 74.4 | 0.392 | 37.65 |
| Nov. 11st | 3.99 | 113 | 677 | 28.1 | 76.3 | 73.6 | 0.295 | 40.89 |
| Nov. 15th | 3.58 | 103 | 730 | 31.1 | 45.0 | 52.0 | 0.117 | 19.68 |
| Nov. 19th | 3.07 | 91 | 552 | - | - | 62.2 | 0.079 | 10.03 |
| Nov. 25th | 3.16 | 98 | 487 | 30.4 | 49.0 | 62.1 | 0.060 | ＜10.00 |
| Nov. 27th | 3.05 | 97 | 471 | - | - | - | 0.070 | ＜10.00 |
| Dec. 4th | 2.75 | 93 | 368 | - | - | - | 0.211 | ＜10.00 |
| Notes: “-” represents undetected, red blood corpuscle (reference range 3.80×10^12^/L～5.10×10^12^/L, the following are the same), hemoglobin (115～150 g/L), platelet (125×10^9^/L～350 ×10^9^/L), albumin (35～50g/L), alanine transaminase (＜40 U/L), aspartate transaminase (＜40 U/L), procalcitonin (0～0.052 ng/ml), C-reactive protein (＜10.0 mg/L) | | | | | | | | |
